# Pest control and resistance management through release of insects carrying a male-selecting transgene

**DOI:** 10.1186/s12915-015-0161-1

**Published:** 2015-07-16

**Authors:** Tim Harvey-Samuel, Neil I. Morrison, Adam S. Walker, Thea Marubbi, Ju Yao, Hilda L. Collins, Kevin Gorman, T. G. Emyr Davies, Nina Alphey, Simon Warner, Anthony M. Shelton, Luke Alphey

**Affiliations:** Department of Zoology, University of Oxford, South Parks Road, Oxford, Oxfordshire OX1 3PS UK; Oxitec Ltd, 71 Innovation Drive, Milton Park, Oxford, Oxfordshire OX14 4RQ UK; Cornell University/NYSAES, Barton Lab 416, 630 W. North Street, Geneva, NY 14456 USA; Institute of Plant Protection, Xinjiang Academy of Agricultural Science, Urumqi, China; Biological Chemistry & Crop Protection Department, Rothamsted Research, Harpenden, Hertfordshire AL5 2JQ UK; Department of Life Sciences, Imperial College London, Silwood Park Campus, Buckhurst Road, Ascot, Berkshire SL5 7PY UK; The Pirbright Institute, Woking, Surrey GU24 0NF UK

**Keywords:** *Bacillus thuringiensis*, Diamondback moth, Insect, Insecticide resistance management, Pest, *Plutella xylostella*, Sterile insect technique, Transgenic

## Abstract

**Background:**

Development and evaluation of new insect pest management tools is critical for overcoming over-reliance upon, and growing resistance to, synthetic, biological and plant-expressed insecticides. For transgenic crops expressing insecticidal proteins from the bacterium *Bacillus thuringiensis* (‘*Bt* crops’) emergence of resistance is slowed by maintaining a proportion of the crop as non-*Bt* varieties, which produce pest insects unselected for resistance. While this strategy has been largely successful, multiple cases of *Bt* resistance have now been reported.

One new approach to pest management is the use of genetically engineered insects to suppress populations of their own species. Models suggest that released insects carrying male-selecting (MS) transgenes would be effective agents of direct, species-specific pest management by preventing survival of female progeny, and simultaneously provide an alternative insecticide resistance management strategy by introgression of susceptibility alleles into target populations. We developed a MS strain of the diamondback moth, *Plutella xylostella*, a serious global pest of crucifers. MS-strain larvae are reared as normal with dietary tetracycline, but, when reared without tetracycline or on host plants, only males will survive to adulthood. We used this strain in glasshouse-cages to study the effect of MS male *P. xylostella* releases on target pest population size and spread of *Bt* resistance in these populations.

**Results:**

Introductions of MS-engineered *P. xylostella* males into wild-type populations led to rapid pest population decline, and then elimination. In separate experiments on broccoli plants, relatively low-level releases of MS males in combination with broccoli expressing Cry1Ac (*Bt* broccoli) suppressed population growth and delayed the spread of *Bt* resistance. Higher rates of MS male releases in the absence of *Bt* broccoli were also able to suppress *P. xylostella* populations, whereas either low-level MS male releases or *Bt* broccoli alone did not.

**Conclusions:**

These results support theoretical modeling, indicating that MS-engineered insects can provide a powerful pest population suppressing effect, and could effectively augment current *Bt* resistance management strategies. We conclude that, subject to field confirmation, MS insects offer an effective and versatile control option against *P. xylostella* and potentially other pests, and may reduce reliance on and protect insecticide-based approaches, including *Bt* crops.

## Background

Pest insects are a major threat to global food production, biodiversity conservation, and human and animal health [1–3]. Synthetic insecticides are widely used for control; however, potential off-target ecological damage, and the capacity of pest populations to develop resistance, has driven demand for alternative methods of pest control. Integrated pest management (IPM) approaches have been developed, utilizing multiple tools including biological insecticides (applied and expressed in transgenic crops), enhanced biological control, mating disruption, and the release of sterile insects (the sterile insect technique, SIT) to sustainably manage insect pest populations.

The SIT reduces target pest populations through sustained mass-releases of radiation-sterilized insects, thus reducing the frequency of mating between fertile insects [4]. SIT relies on the mate-seeking and mating behavior of released insects, and is therefore species-specific and can be effective against pests that are difficult to control by other methods. SIT has been successful in area-wide eradication and suppression programs against numerous crop pests [5]. Wider applicability of SIT is hindered by several challenges, including the negative effects on insect performance of sterilization by irradiation [6–10] and difficulty in conducting large-scale sex-sorting for male-only releases [11–13].

We have previously developed a male-selecting (MS) transgenic system to overcome these obstacles [14, 15]. In this system, pest colonies are engineered with tetracycline-repressible dominant female-specific lethal transgenes. Provision of tetracycline (or suitable analogues) to larval stages suppresses transgene lethality allowing mass rearing. Once released, mating between transgenic and wild insects results in mortality of female progeny (female-specific lethality) due to the absence of suitable quantities of tetracycline in the field, thereby reducing the reproductive potential of the target population [15–20]. Through targeting female progeny but allowing male transgene heterozygotes to survive to reproduce, the MS system is predicted to be significantly more efficient at suppressing populations than those which target both sexes (such as SIT) [19]. Additionally, this system avoids the negative impacts of irradiation on released insect competitiveness [21] and enables large-scale (off tetracycline) production of single-sex (male) release cohorts. Male-only releases can significantly improve per-male efficiency [22, 23] by concentrating the reproductive effort of released insects on wild females. For the Mediterranean fruit fly (medfly, *Ceratitis capitata*), SIT programs have, in tandem with sterilization by radiation, relied on translocation-based sex-sorting systems in which a dominant marker is translocated to the Y chromosome [24]. However, these traits are difficult to translate to new pest species, are unstable, and compromise insect productivity in mass rearing [25, 26].

The release of male insects carrying MS transgenes (“MS males”) has been shown to be effective in suppressing target pest populations in cage experiments against the mosquito *Aedes aegypti*, *C. capitata*, and the olive fly (*Bactrocera oleae*) [18, 20, 27]. However, the pest suppression potential of such a transgenic system has not yet been investigated in lepidopterans, which include many of the most destructive pests of forestry and agriculture worldwide [28]. Beyond this direct population-reducing effect, modeling suggests that releases of MS males into a target population may simultaneously provide an insecticide resistance management benefit. Mating between released males and wild females results in the survival of male transgene heterozygotes and the introgression of their background genetics into the wild pest population [29, 30]. With an insecticide-susceptible genetic background in released insects, this introgression will increase the frequency of susceptibility alleles within the target pest population.

This proposed mechanism of resistance management is analogous to that currently utilized in transgenic crops engineered to express insecticidal Cry toxin proteins from the bacterium *Bacillus thuringiensis*. A major advantage of these ‘*Bt* crops’ is their low environmental impact, with the effects of the toxin limited to target species both taxonomically (due to the high species-specificity of *Bt* toxins) and ecologically (as toxin expression is limited to crop tissue, which needs to be ingested to take effect). Cultivation of *Bt* crops, which primarily target lepidopteran and coleopteran pests, has increased rapidly over the past two decades, reaching 78.8 million hectares in 2014 [31]. Resistance in pest populations is an ongoing threat to transgenic *Bt* crop efficacy [32, 33]. The most widely applied resistance management strategy is known as high dose/refuge [34]. Here, the *Bt* toxin is expressed at sufficiently high levels for resistance to be functionally recessive and a proportion of the crop grown includes non-*Bt* varieties (the refuge). As with modeled MS-based resistance management, refugia therefore act as a source of susceptible alleles which introgress (via mating) into the pest population*,* reducing the frequency of *Bt*-resistant homozygotes. Although the high-dose/refuge strategy has been largely successful in delaying *Bt* resistance, the development of *Bt*-resistant populations has now been reported in the field, particularly in lepidopteran pests (reviewed in [32]). Additionally, recommendations on refuge size vary considerably depending on the cultivated species and the number of *Bt* transgenes expressed, but may be as high as 50 % of the crop, potentially exposing large areas to economic levels of damage [35]. Novel means of delaying *Bt* resistance, especially those which would function as effective pest control measures in their own right, would therefore be of economic and ecological benefit.

Models of population genetics and dynamics predict that release of MS males carrying insecticide susceptibility alleles could form effective components of insecticide resistance management (IRM) strategies for *Bt* crops [29, 30]. Such releases could substantially reduce refuge size requirements for equivalent levels of resistance management, or make refuges redundant altogether, as well as providing a potential remedial action to reverse the spread of resistance where present. Furthermore, the overall population suppression benefit of an integrated program combining *Bt* plants and MS insects is anticipated to be better than either alone. These effects are predicted at release rates significantly lower than those usually employed for population control in SIT programs.

Testing resistance management systems for *Bt* crops requires an insect that has evolved resistance to *Bt* proteins and plants that express such proteins. Diamondback moth, *Plutella xylostella*, is a major pest of brassica crops costing an estimated US$4-5 billion annually in losses and control costs worldwide [36]. *P. xylostella* was the first agricultural insect pest to have evolved resistance in the field to *Bt* proteins [37, 38]. Of the >500 insecticide-resistant arthropods documented, *P. xylostella* ranks second in the number of cases of resistance and is considered one of the most difficult insect pests to control [39]. Novel means of controlling this highly resistant pest are thus required [40]. Brassica plants have been developed that express *Bt* proteins [41–45] and the combination of *P. xylostella* and *Bt* brassica plants has served as an effective model system for studying resistance management of *Bt* proteins expressed in plants [46, 47]. We have developed a MS system in *P. xylostella* potentially suitable both as a novel control tool for growers, and as a model system for testing the predicted benefits of MS insect releases for managing resistance to *Bt* and other insecticides [15]. A strain of *P. xylostella* transformed with this system, called OX4319L, shows tightly controlled, highly penetrant female-specific lethality [15] and is sexually competitive against non-transgenic individuals [48]. Herein, we describe experiments that demonstrate the direct population-suppressing and *Bt* resistance management effects of releasing OX4319L males into wild-type populations.

## Results

### Population suppression

**Fig. 1:**
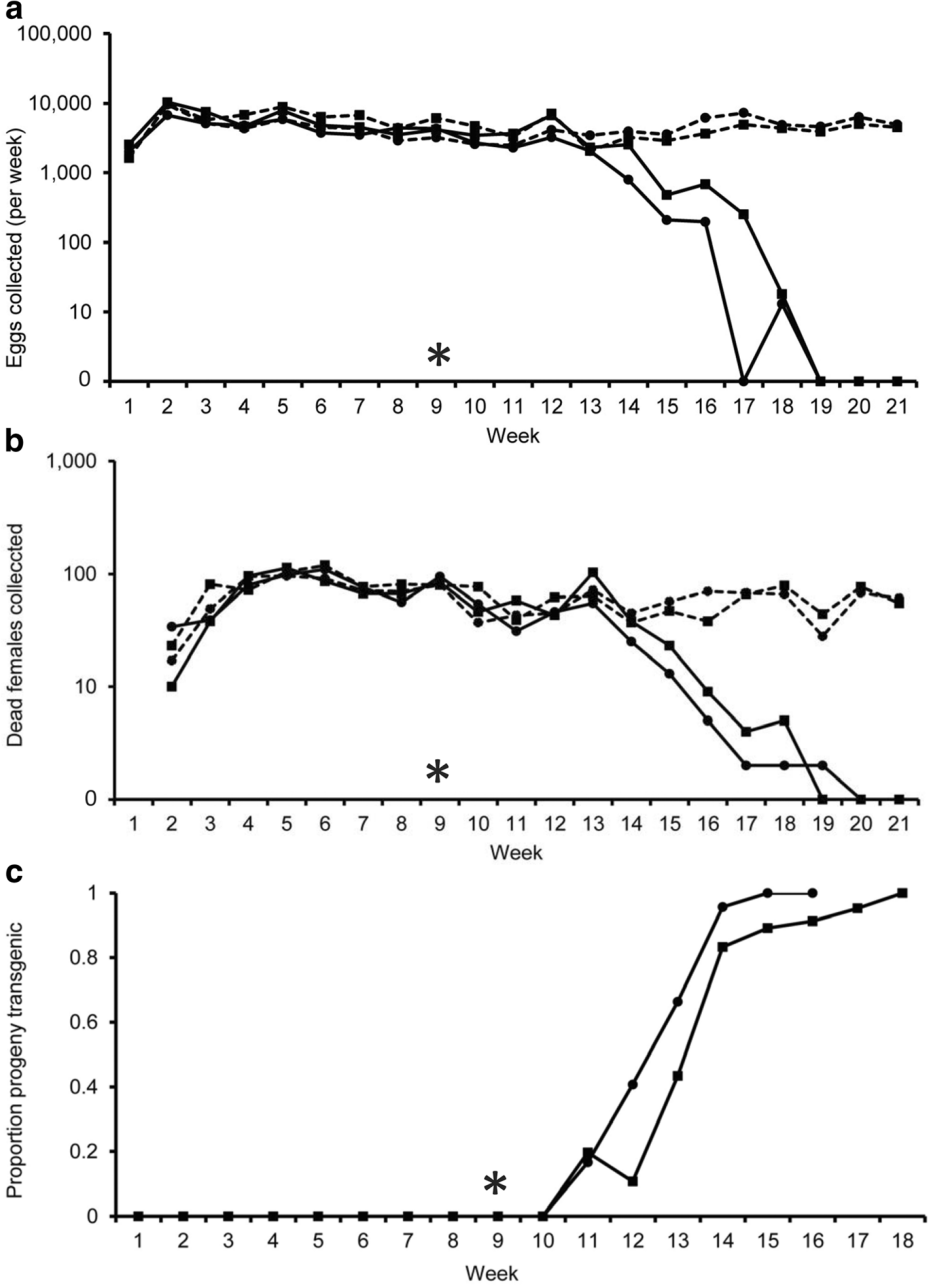
Suppression of caged populations of *Plutella xylostella* by weekly introduction of OX4319L males. Graphs showing (**a**) number of eggs collected, (**b**) number of dead adult females collected weekly from cages, and (**c**) proportion of cage progeny that were transgenic re-entering the cages (fluorescence proportions), over the experimental period. With female moths present in the cages being only wild-type, transgenic progeny (including those re-introduced) were heterozygotes (restrictive conditions). Fluorescence proportions thus equate to twice the MS transgene allele frequency in the cage population at that time point. Solid lines represent OX4319L-treated populations (Cages 1 and 2, circular and square data-points, respectively). Dashed lines represent untreated control populations (Cages 3 and 4, circular and square data-points, respectively). In week 9, return of pupae into treatment and control cages was made proportional and release of OX4319L males into treatment cages began (marked with asterisk)

This experiment investigated the direct population suppression potential of the OX4319L MS transgene-carrying *P. xylostella* strain, independent of other control measures. Releases of transgene-homozygous OX4319L males into two experimental cages began 9 weeks after the initial wild-type introductions into the cages. At this point the population size, estimated by weekly consistency of egg production, in each cage was judged to have reached equilibrium (Fig. 1a). The first re-introductions of transgenic progeny (as pupae, evidenced by positive screening of the DsRed2 fluorescent transgene marker) into the two treatment cages took place 2 weeks later (Fig. 1c), indicating successful mating by OX4319L males. The proportion of re-introduced pupae that were transgenic (fluorescence proportion) increased as releases continued into the treatment cages, eventually reaching 100 % 7 and 9 weeks after OX4319L releases began. Under the restrictive (non-tetracycline) conditions of this experiment, the transgenic (fluorescent) phenotype was restricted to a single genotype (male heterozygotes) as female transgene carriers are unable to survive to adulthood [15]. Transgenic males were therefore only able to mate with wild-type females and the fluorescence proportions recorded here are equal to twice the transgene allele frequency in the population [48]. The increasing introgression of MS transgenes into these treatment populations had a substantial effect on the population sex ratio and reproductive capacity. By week 15 (6 weeks after OX4319L releases began) the number of dead adult females collected in each of the treatment cages had decreased considerably relative to that of control cages (Fig. 1b), concurrent with a reduction in the reproductive output in these cages (Fig. 1a). As the generation time (egg to egg) of the insects in these experiments was approximately 3 weeks, these time periods (between initiation of MS male releases and suppression, approximately two generations) fit the hypothesis that introductions of the OX4319L transgene into the treatment populations were causing reductions in the number of females reaching adulthood, and thus the number of eggs being laid in the subsequent generation. Ten weeks after OX4319L releases began (week 19) the reproductive output of both treatment cages had dropped to 0, and no dead female moths were collected after this point. In this experimental protocol this equates to approximately three generations. Experiments were continued for another 2 weeks after egg-laying ceased, confirming that their populations were extinct.

### Insecticide resistance management

This experiment investigated the predicted benefits to both resistance management and population control of combining *Bt* transgenic crops and MS males with *Bt*-susceptible genetic backgrounds. To achieve this, treatments were designed such that each pest control method on its own (*Bt* only and OX4319L release at low levels) would likely be insufficient for population control. The effect of the combination of treatments could then be compared with that of each one applied singly. In the *Bt* only treatment, the presence of resistance alleles in the founder population, and strong selection for these alleles in subsequent generations, were expected to result in a lack of effective control. In the low release rate OX4319L-only treatment, this was achieved by selecting a release rate predicted to be unable to prevent population growth under these conditions. Under this design, all treatment populations would persist for the duration of the experimental period, allowing comparison of their population densities and resistance allele frequencies after multiple generations of treatment effects. In addition, a treatment where OX4319L males were released at a high rate (in the absence of *Bt*) was also conducted to act as an investigation of this strain’s suppression potential under more challenging conditions where the target pest population may be expanding rapidly.

**Fig. 2:**
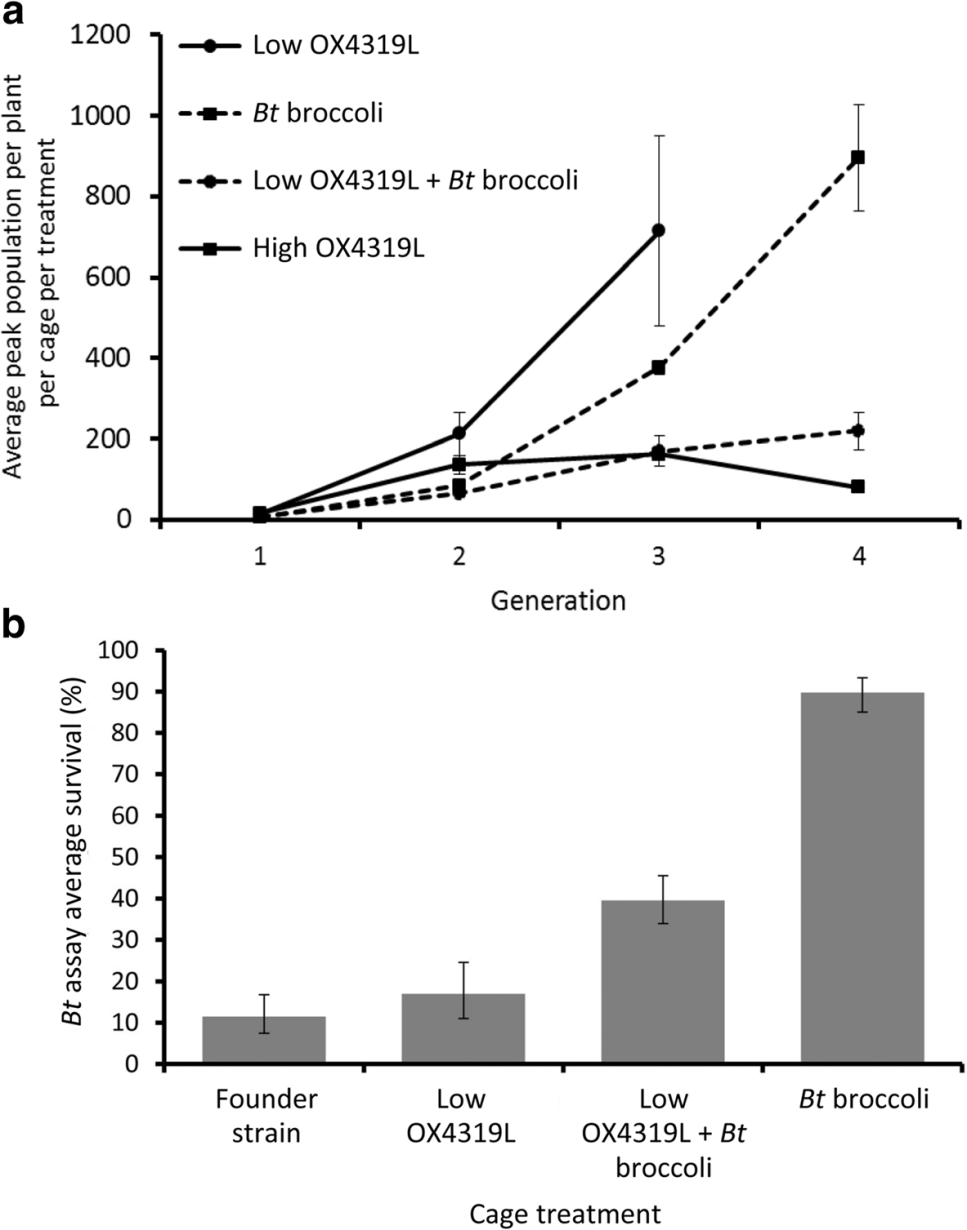
Effects of *Bt* broccoli and OX4319L releases on caged *Plutella xylostella* populations over multiple generations. Caged populations were established with hybrid *Bt*-resistant/wild-type insects – the founder strain – with a low estimated frequency of homozygous-resistant individuals. **a** Graph shows the mean peak population size per plant, per generation, in four experimental treatments over the experimental period: Treatment 1, *Bt* broccoli, no OX4319L releases; Treatment 2, *Bt* broccoli, low-rate weekly OX4319L releases (release rate of 3:1 in Generation 1, increased to 5:1 in subsequent generations); Treatment 3, non-*Bt* broccoli, low-rate weekly OX4319L releases (identical release rates to Treatment 2); and Treatment 4, non-*Bt* broccoli, high-rate weekly OX4319L releases (release rate of 20:1 in Generation 1, increased to 40:1 in subsequent generations). Means were calculated from three experimental cage replicates, with the exception of Treatments 2 and 4, which were reduced to two and one cage replicates in Generation 3, respectively. Treatment 3 cages were terminated in Generation 3 as the insect populations had reached maximum capacity. Error bars represent standard error of the mean. **b** *Bt* survival assays. Mean survival of third-instar larvae from three experimental cage treatments and the founder strain used to begin these experimental treatments when exposed to a discriminating dose of *Bt* in artificial diet assays. *Bt* dose in this assay is high enough to ensure that only homozygous *Bt*-resistant individuals will survive (as in the high-dose/refuge strategy). This assay therefore indicates the proportion of each population remaining *Bt*-resistant (homozygous) and *Bt*-susceptible (heterozygous or homozygous-susceptible). For each cage, two *Bt* assays and one no-*Bt* control assay were performed. *Bt* assays in each cage were summed and means represent averages of each set of treatment cages corrected for control mortality. The assays took place using individuals from the final generation in which each treatment was run or, in the case of the founder strain, in the generation prior to the start of the experiment. Survival was corrected for control mortality prior to analysis and error bars represent Pearson’s exact confidence intervals. Survival of insects from the low OX4319L release cages (Treatment 2) was not significantly different from the founder strain; other pairwise comparisons are significantly different (Table 1)

In cages with *Bt* broccoli and no OX4319L releases, *P. xylostella* populations were well-controlled in Generation 1 (Fig. 2a), presumably due to the initially high frequency of genotypes susceptible to the *Bt* toxin. The subsequent populations in these cages, now highly *Bt*-resistant due to the effects of strong selection in the initial generation, then increased rapidly until Generation 4. Similarly, as expected, low-level releases of OX4319L males into *P. xylostella* populations reared on non-*Bt* broccoli were not effective at preventing population growth, and this treatment was terminated at Generation 3. When *Bt* broccoli and low OX4319L releases were combined, however, the caged populations were well-controlled throughout, only increasing slowly at each generation. In Generation 3, the mean peak population counts recorded for the *Bt* broccoli-only and the low-rate OX4319L-only treatments did not differ significantly (Contrast 1). Concurrently, the mean peak population counts in the combined OX4319L + *Bt* broccoli treatment were significantly lower than those of the low-rate OX4319L-only treatment (Contrast 2), but not significantly different to that of the *Bt* broccoli-only treatment (Contrast 3) (Contrast 1: diff = 337, *P* = 0.133; Contrast 2: diff = −545, *P* = 0.0272; Contrast 3: diff = −207, *P* = 0.315). By Generation 4, however, populations in the *Bt* broccoli-only treatment were significantly larger than those where *Bt* broccoli was combined with low-rate OX4319L releases (t = −4.84, *P* = 0.0084). The high rate of OX4319L releases performed similarly to the combined OX4319L + *Bt*-broccoli treatment, but by Generation 4 had started to reduce the population in the last remaining cage. Due to the lack of replication in this treatment in Generation 4, it was excluded from statistical analysis.

Larval *Bt* survival assays conducted in the last generation of each treatment, and on the founder strain in Generation 0, showed a significantly reduced proportion of resistant individuals in treatments that combined *Bt* broccoli and low-rate OX4319L releases compared to those where *Bt* broccoli was used alone (Fig. 2b). Survival rates on *Bt* were low and not significantly different between the founder strain (11.5 %; CI, 7.4–16.8 %) and the low-rate OX4319L-only caged populations (17 %; CI, 11–24.5 %; Table 1). Both treatments in which larvae were exposed to *Bt* selection (Treatments 1 and 2) displayed significantly higher levels of survival on *Bt* compared to those where non-*Bt* broccoli plants were used, indicating high levels of selection against susceptible genotypes in these cages. However, significant differences between treatments with *Bt* plants were apparent. In the *Bt* broccoli-only treatment, *Bt* resistance rapidly increased in frequency in the population, as indicated by the high percentage survival rate (89.7 %; CI, 85–93.3 %). In the combined OX4319L and *Bt* broccoli treatment, the survival rate was significantly lower (39.5 %; CI, 33.9–45.5 %), implying a significantly higher frequency of *Bt*-susceptible alleles (and thus individuals subject to *Bt* selection pressure) in the populations where low-level releases of OX4319L males had taken place.

**Table 1:** Pairwise comparisons of survival assay data (from experiments described in Fig. 2b)

Comparison		Z value	*P* value
Founder strain	Low OX4319L	1.212	0.605
Founder strain	Low OX4319L + *Bt* broccoli	5.016	<0.001
Founder strain	*Bt* broccoli	8.557	<0.001
Low OX4319L	Low OX4319L + *Bt* broccoli	3.323	0.0048
Low OX4319L	*Bt* broccoli	−6.682	<0.001
Low OX4319L + *Bt* broccoli	*Bt* broccoli	−5.594	<0.001

Output was generated using an omnibus logit model for categorical data analysis, followed by post-hoc analysis using Generalized Linear Hypothesis Testing

Allele frequencies derived from these mean survival assay results indicated that the *Bt* resistance allele had increased in frequency over the experimental period in populations under *Bt* selection, from 0.36 in Generation 0 (founder strain) to an estimated 1.0 in the *Bt* broccoli treatment and 0.71 in the combined OX4319L and *Bt* broccoli treatment (both Generation 4). Fluorescence proportions determined for treatments in which OX4319L males were introduced estimated that 56.0 % (CI, 50.4–61.5 %) of the combined OX4319L and *Bt* broccoli treatment populations (Generation 4); 55.6 % (CI, 47.2–63.7 %) of the low OX4319L treatment populations (Generation 3); and 87.5 % of the high release rate OX4319L treatment population (Generation 3 – single replicate) carried the MS transgene. As in the population suppression experiment, these fluorescence proportions are equal to twice the MS transgene allele frequency in these populations [48].

## Discussion

We found that sustained releases of male *P. xylostella* moths carrying a transgene which allows only males to survive to adulthood can have a direct suppressing effect on a target pest population, as previously shown for dipteran pests [18, 20, 27]. We also demonstrate that releases of such insects can significantly delay the spread of resistance to *Bt* in target pest populations by introgression of susceptibility alleles through male progeny, even when initial resistance levels are high. With the increasing number of cases of field resistance to *Bt* plants, transgenic systems such as the one examined here may play an important role in IRM programs aimed at delaying or overcoming the evolution of resistance to this valuable technology.

In stable target populations reared on artificial diet and an initial OX4319L over-flooding ratio of approximately 10:1, reduced numbers of females and suppression of the populations’ reproductive capacity became evident approximately two generations after OX4319L male releases were initiated and these populations fell rapidly to extinction thereafter (after approximately three generations). This timeframe until extinction compares well with previous cage experiments exploring the pest suppression potential of female-lethal transgenes in *A. aegypti* (4.66 ± 0.88 mean generations until extinction; 8.5–10:1 over-flooding rate) [27], *B. oleae* (3 ± 0.0; 10:1) [18], and *C. capitata* (3 ± 0.0; 10:1) [20]. With such MS strains applied against isolated populations, as reported here, local eradication of the target pest is achievable as the over-flooding ratio increases as the target population falls. This effect would be lessened when applied in the field against non-isolated pest populations or when pests migrate between areas. However, even though *P. xylostella* is known to travel large distances in some circumstances [49–52], it does not typically travel far when host plants are available [53, 54]. Although complete isolation of *P. xylostella* and other pest species would be unlikely in most scenarios, efficient control of target populations is likely achievable with some degree of population isolation and/or with sufficient scale of application to reduce edge effects. Moreover, the SIT has previously demonstrated efficient control of other lepidopteran pests – codling moth (*Cydia pomonella*) and pink bollworm (*Pectinophora gossypiella*) – that are able to travel relatively large distances [55, 56].

In our experiments on broccoli, target populations were free to expand in protected environments devoid of limiting factors such as predators and adverse climatic effects. Here, OX4319L male release, with a high over-flooding ratio of up to 40:1, maintained a high level of control. As expected, low rates of OX4319L male releases alone – over-flooding ratio of up to 5:1 – did not prevent rapid population growth under these conditions. Whether a management strategy is able to prevent the expansion of a pest population is a function of that strategy’s ability to counteract the reproductive rate (*R*_0_) of the target species [57]. In the case of the lower rate OX4319L treatment, the proportion of matings won by these released insects was predicted to be low enough that the population’s reproductive capacity would remain well above 1. Pests with a high *R*_0,_ such as Lepidoptera, can represent a challenge to mating-based pest management approaches, and in some cases higher release rates may be required to counter this. However, in a controlled greenhouse setting, *R*_0_ is artificially elevated, while in the field, mortality rates due to parasitism, predation, pathogen infection and environmental factors, especially in pre-adult stages, are likely to be high [40]; it is possible that these factors would reduce the release rate of OX4319L males required to control a target population, relative to release rates used in these experiments.

Populations maintained on *Bt* broccoli alone expanded rapidly after strong selection for *Bt* resistance. However, low rates of OX4319L male release in combination with *Bt* broccoli significantly reduced population growth, controlling the target populations well when each treatment on its own failed to do so. This apparent synergistic effect supports model predictions that introgression of susceptibility alleles into a target population by released transgenic males could prevent or reverse the spread of resistance, thereby preserving susceptibility to *Bt* toxins [29, 30].

Consistent with the hypothesis underlying these models, results from our larval *Bt* survival assays showed significantly higher survival (frequencies of resistant homozygotes and, consequently, resistance alleles) in the *Bt* broccoli-only treatment compared with cages where low levels of OX4319L males were introduced in addition to *Bt* plants. Mean fluorescent protein marker frequencies in these combined OX4319L + *Bt* broccoli cages (56.0 %) displayed a high level of agreement with the proportions of these populations which were *Bt*-susceptible, once survival assay results were calibrated for homozygous-resistant mortality (100–43.7 = 56.3 %). These results are what would be expected if mating by released MS males were the source of *Bt*-susceptibility alleles in these populations, diluting the frequency of resistant (homozygous) genotypes and counteracting *Bt* resistance at a rate predicted by their mating success. Under this hypothesis, no such agreement would be expected if resistance alleles were not undergoing positive selection in the target population, as pre-existing susceptibility alleles would persist independent of those introgressed by MS males. Supporting this argument, populations treated with low-dose OX4319L releases, which received identical release rates of transgenic males to the low OX4319L + *Bt* broccoli treatment (resulting in a very similar population-level fluorescence ratio, 55.6 %) but were not exposed to *Bt* selection, showed a level of *Bt* susceptibility independent of their fluorescence proportions (mean proportion of individuals not surviving *Bt* assay = 100–18.4 = 81.6 %). Taken together, these results provide an empirical demonstration of the mechanism by which MS transgenic insects carrying insecticide-susceptible genetic backgrounds can maintain insecticide efficacy, even in the face of continuous, strong selection for resistance.

These resistance management effects were explored using a well-established model system utilized for investigating IRM strategies for *Bt* crops and risks to non-target organisms [58–60]. Thus, although *Bt* crucifers are not yet commercially cultivated, the mechanism we demonstrate is broadly relevant to *Bt* crops. In addition, depending on the nature and inheritance of resistance, similar effects would be expected for *Bt* sprays or indeed any other insecticidal intervention against *P. xylostella* and other pests. The efficiency of a transgenic strain in providing these effects will likely depend on performance of its males in the field. Previous studies on OX4319L indicate that this transgene insertion is associated with a small fitness cost in the laboratory [48], but its field performance has not yet been tested.

Theoretical population modeling studies have investigated the release of fertile non-transgenic insecticide-susceptible insects into *Bt* crop areas [29]. At present, the only feasible option for achieving sex separation in lepidopterans, at the scales required for release, is the MS system described herein, so in practice non-transgenic releases would comprise both males and females. In these models, introgression of susceptibility alleles from such releases also provided a degree of resistance management. However, released fertile females would lead to higher pest populations than MS releases, at least initially, and may necessitate increased insecticide usage in neighboring conventionally sprayed areas. For these reasons, an IRM program involving releases of fertile pest females would likely be unacceptable to growers and regulators.

The release of radiation-sterilized *P. gossypiella* has also been described as preventing development of resistance to *Bt*-expressing cotton [61]. However, these two systems (SIT and MS) provide fundamentally different ways of managing resistance. Whereas SIT may prevent increasing resistance by killing resistant insects before they have a chance to reproduce (any alternative population-suppression method would have the same effect), the MS phenotype additionally manages resistance by actively introgressing susceptibility alleles into the target population through the survival of male progeny. In areas of 100 % *Bt* crop cultivation these effects on resistance management may be similar, albeit with these benefits likely to be provided at lower release rates using MS males [22, 23]. However, if combined into existing *Bt*-IRM programs, MS releases may also be advantageous to the maintenance of susceptible populations within refuges as male MS heterozygotes within these areas will survive and continue to pass on susceptibility alleles in subsequent generations. Although some suppression of these refuge areas is expected under these conditions, our results demonstrate that, in the absence of other control measures such as *Bt*, low-level releases of MS males will have a minimal effect on population growth. Moreover, this mechanism may be of benefit to growers utilizing sprayed insecticides as introgressed susceptibility alleles will be spread through the natural mating behavior of the pest population between insecticide sprays. Future modeling studies may help to elucidate the relative benefits of these two potential alternatives to refuges in combating resistance to *Bt*.

## Conclusions

The results described herein demonstrate that releases of MS transgene-carrying, insecticide-susceptible insects can provide two simultaneous effects on pest populations – direct population suppression through death of female progeny and IRM by introgression of susceptibility alleles into target populations through surviving male progeny. Further laboratory experiments are required in order to disentangle the relative contributions of these two effects in suppressing pest populations on *Bt* plants. Nonetheless, our findings strengthen the argument that MS systems, such as OX4319L, could function as effective stand-alone tools against given pest species or as a highly compatible component of an integrated pest management program. If used as an alternative to broad-spectrum insecticides within such an IPM framework, the species-specific action of MS transgenes would reduce negative effects on non-target organisms including natural enemies which can further delay the evolution of resistance to *Bt* crops [60, 62, 63]. For successful IPM programs against *P. xylostella*, conservation of these natural enemies is important [40] and can provide more effective control than the use of broad-spectrum insecticidal sprays [64]. These conservation benefits are also likely to apply to other groups such as pollinators and birds which are currently threatened by the use of some agricultural insecticides [65].

Existing recommendations on minimum refuge size in some *Bt* crops, and compliance with these recommendations, may be inadequate for robust resistance management [32, 35]. MS insects carrying *Bt*-susceptible genetic backgrounds could help to reduce refuge requirements, or potentially replace or supplement refugia as a resistance management tool in *Bt* crops [29, 30]. Area-wide releases of transgenic MS insects in place of refugia could offer area-wide protection to growers and seed producers, for whom such compliance can be expensive and impractical. In addition, our results demonstrate that use of MS systems in combination with other pest management methods would reduce the required number of insects to be produced and released to achieve a given level of control.

The utility of the MS system has been demonstrated here in a lepidopteran pest and elsewhere in dipteran pests [15, 16, 18, 20, 66], so further development in pests in these and other taxonomic groups appears feasible and should be evaluated. This technology represents a promising and sustainable pest management tool offering significant benefits to future IPM strategies.

## Methods

**Fig. 3:**
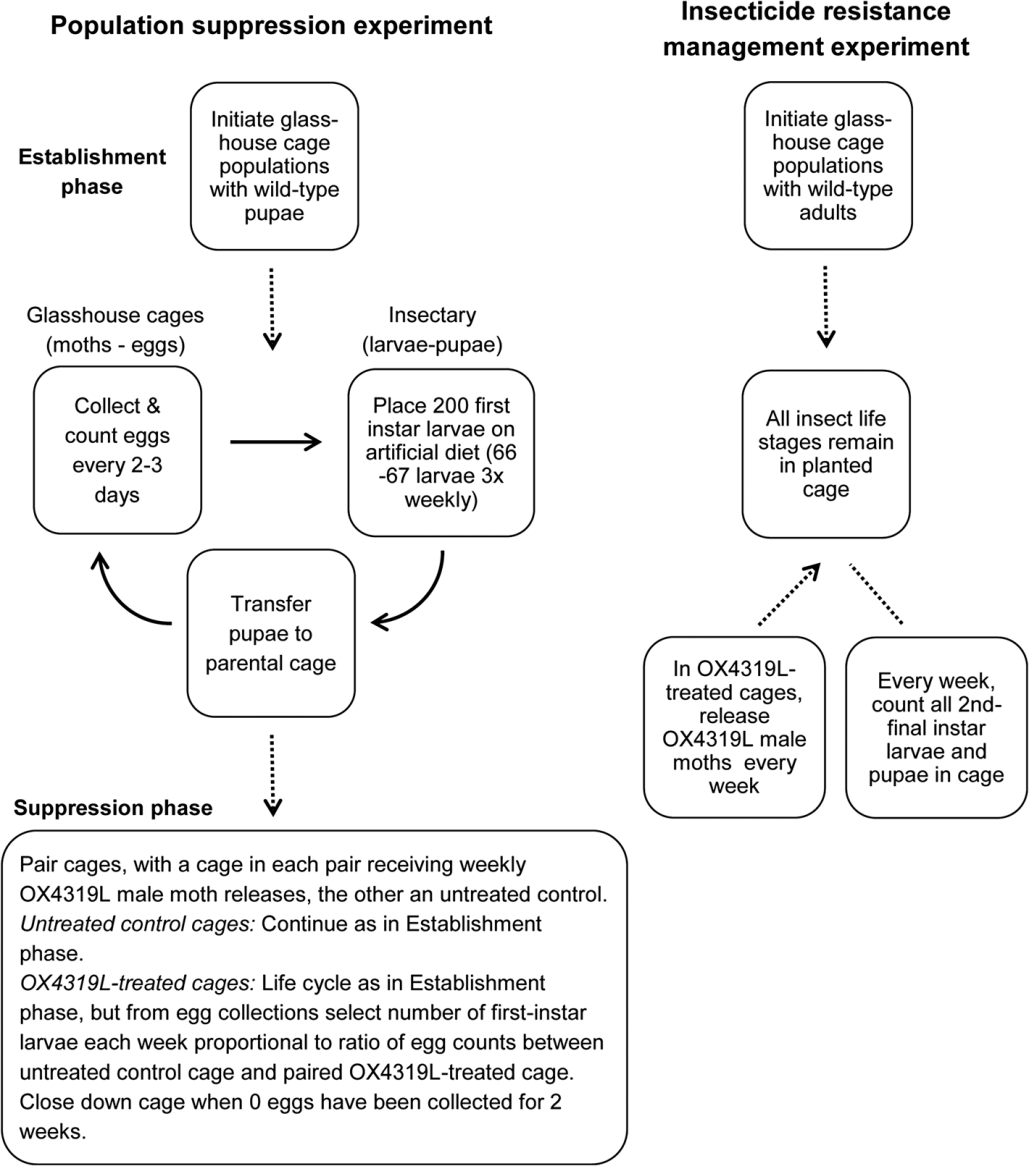
Schematic showing design of the population suppression and insecticide resistance management experiments

A summary of the two experimental designs used is provided in Fig. 3. More detailed explanations of these designs and their aims are provided below.

### Summary of experimental designs

The glasshouse cage population suppression experiment was designed to simulate a field scenario in which biotic and abiotic mortality factors maintain a stable pest population, with adult moths mating and laying eggs in glasshouse cages, and larval rearing being conducted in a separate temperature-controlled room [18, 20, 27]. When weekly OX4319L male moth releases started, OX4319L-treated cages were paired with an untreated counterpart (control cage) and the number of pupae subsequently reintroduced into each treatment cage was made proportional to the ratio of eggs counted between the treatment cage and its paired control cage: this allowed the expected reduced number of females in OX4319L-treated cages to be reflected in reduced numbers of pupae entering the population in the next generation.

In contrast, the subsequent glasshouse cage IRM experiment was conducted on broccoli plants – some on *Bt* broccoli – as they offered a realistic model for many agricultural systems with transgenic crops. Artificially maintaining stable populations on plants, by accurately manipulating the number of progeny surviving, was deemed impractical, and the populations were therefore allowed to expand freely in the near-absence of biotic and abiotic mortality factors, with all life stages of each experimental population residing in their respective cage. This second experiment provided a more rigorous test of the pest management potential of the OX4319L strain as the intrinsic growth rate of target populations was not artificially controlled and competitors from these target populations had the advantage of being reared on host plant material, as opposed to an artificial diet medium.

### Experimental population structures

Generations in the population suppression experiment were continuous while those in the resistance management experiment were discrete, at least within the experimental period. In the population suppression experiment a continuous generational structure was achieved by introducing pupae into cages three times per week. As the lifecycle of *P. xylostella* in this experiment was 3 weeks, this created a stable, mixed-age adult population with eggs being collected every 2–3 days over the experimental period. In the resistance management experiment conducted on broccoli, populations were founded by a single introduction of adult moths. Adults in this founding generation, and those in subsequent generations, were allowed to mate freely in their respective cages with females from the same generation ovipositing over a number of days. As such, within the experimental period generations remained discrete and were discernable by peaks in weekly population counts which, as expected, reached a maximum level once per generation (approximately every 3 weeks; see below). Generations did, however, begin to overlap over time.

The generation time of *P. xylostella* in the insecticide resistance experiment was approximately 20–23 days (incrementally: egg, 3–4 days; larvae, 13–14 days; pupae, 3–4 days; adult time to sexual maturity, 1 day). As experiments were conducted in a glasshouse, these time periods varied depending on outdoor weather conditions.

### Population suppression experiment

Experiments were conducted in quarantine facilities at Rothamsted Research, Hertfordshire, UK in accordance with legislation concerning the contained use of GM organisms in the UK. Larval rearing took place in a temperature-controlled room and experimental cages were housed within a temperature-controlled glasshouse (both 25 °C and 16:8 light:dark photoperiod). As an efficient, easily replicable proxy for host crop infestation, larval rearing was conducted on artificial diet medium and followed the methods of Martins et al. [67]. All experimental populations were reared on non-tetracycline diet. Transgene-homozygous OX4319L males were produced on diet with chlortetracycline hydrochloride (100 μg/mL) and sexed manually prior to introduction. Experimental cages measured 120 × 100 × 120 cm (width × depth × height), with a zipped entrance at the front. Insects were added to the cages as pupae.

The experiment comprised two phases: establishment and suppression. During establishment, stable mixed-age populations of wild-type *P. xylostella* (wild-type background strain to OX4319L isolated in Vero Beach, Florida, USA) were established in each of the four cages. During suppression, weekly introductions of transgene-homozygous OX4319L males were made into two of the cages to investigate whether engineered female-specific lethality resulted in suppression of these populations. Throughout the experiment, cabbage-extract-baited Parafilm (Bemis Company Inc., Oshkosh WI, USA) pieces were hung from the roof of the cage to act as an artificial leaf oviposition substrate. These were replaced three times per week (Monday, Wednesday, and Friday) and eggs collected on each sheet were counted. During each egg collection, dead adult moths and uneclosed pupae were also collected from the cages, sexed, and counted. Eclosed adults were provided with sugar water-saturated cotton wool reservoirs, changed every 2–3 days.

### Establishment phase

Cage populations were initiated by placing 200 unsexed wild-type pupae into each cage. Stable non-expanding populations were maintained in each cage to mimic the stabilizing effects of predation and other limiting factors in the wild. This was achieved by introducing a constant number of pupae back into the cages each week. A total of 200 first-instar larvae were selected each week to carry on the population, taken from the three weekly collections (67 larvae chosen from Monday collection, 67 from Wednesday, and 66 from Friday). These larvae were reared in plastic beakers on non-tetracycline diet, and after pupation were sexed and transferred back into the cage from which they had been collected as eggs, 2 weeks earlier. Tri-weekly egg collections (and subsequent tri-weekly pupal reintroductions) maintained stable, mixed-age populations as might be expected in the field, where adult moths would be continuously entering the population. The first two introductions of wild-type moths into each cage (weeks 1 and 2) originated from an independent laboratory colony. From this point onwards each cage population was self-sustaining.

### Suppression phase

Once egg counts had stabilized (indicating stable populations) in week 9, two of the four cages were chosen as OX4319L treatment cages (Cages 2 and 4) and two as control cages (Cages 1 and 3). Cages were designated in a blocked design to minimize bias caused by uncontrolled abiotic factors. In non-treatment control cages, the protocol from the establishment phase was continued. Each OX4319L treatment cage was randomly paired with a control cage for the remainder of the experiment (Cages 1 and 2; Cages 3 and 4). The reintroduction of pupae into each OX4319L treatment cage followed the same protocol as for the establishment phase; however, the total number of early-instar larvae selected for rearing that week was made proportional to the ratio between the number of eggs collected for that treatment cage and its paired control cage for the week when these larvae were collected as eggs. This method ensured that the population suppression effect of female death of transgenic larvae, reflected later by reduced number of eggs collected, resulted in reduced numbers of pupae re-entering the OX4319L-treated cages relative to an untreated population.

After cages had been paired, weekly introductions of homozygous OX4319L males into the treatment cages began. The target over-flooding ratio (OX4319L to wild-type males entering the population) was set at 10:1. This release rate is in line with previous studies investigating the effect of female-lethal transgenes on caged populations [18, 20, 27] and much lower than the sterile:wild ratios that successful SIT programs have aimed to achieve against other Lepidoptera: *P. gossypiella* (60:1) [68]; *C. pomonella* (40:1) [69]; and painted apple moth, *Orgyia anartoides* (100:1) [70, 71]. This over-flooding ratio was calculated as 10× the mean male recruitment rate for the 3 weeks preceding OX4319L male introduction, and the numbers released were held constant for the remainder of the experiment (Cage 2: 990 OX4319L males; Cage 4: 980 OX4319L males). Males were released as pupae into the cages once per week (Wednesday).

Pupae reintroduced into treatment cages were screened for the DsRed2 fluorescent protein transformation marker and fluorescence proportions (proportion of the population which carried the transgene) recorded. Under the restrictive conditions of this experiment and the highly penetrant female-lethal phenotype of OX4319L [15], population-level transgene allele frequencies were equal to half the fluorescence proportion [48]. Each treatment cage’s end-point – extinction of each population – was pre-defined as two consecutive weeks of zero eggs collected.

### Insecticide resistance management (IRM) experiment

Experiments were conducted at Cornell University, New York State Agricultural Experiment Station, Geneva, NY, in accordance with legislation concerning the contained use of GM organisms in the USA. All *P. xylostella* experimental populations in this experiment were reared on broccoli plants and allowed to expand freely. Eleven cages, each 1.83 m × 0.91 m × 1.83 m (length × width × height), were placed in a temperature-controlled glasshouse with supplementary lights (23–27 °C, 16:8h light:dark photoperiod, and uncontrolled relative humidity). The 11 cages were assigned to four treatments: Treatment 1, *Bt* broccoli plants, no OX4319L release; Treatment 2, *Bt* broccoli plants, low-rate weekly OX4319L releases; Treatment 3, wild-type plants, low-rate weekly OX4319L releases; and Treatment 4, wild-type plants, high-rate weekly OX4319L releases. Treatments 1–3 were assigned three cages (replicates) while Treatment 4 was assigned two cages.

### Experimental *Plutella xylostella* strains

Two strains were used: a hybrid *Bt*-resistant/wild-type strain used to generate starting populations in the cages (founder strain) and OX4319L. To generate the founder strain, 25 males from a homozygous Cry1Ac *Bt* toxin-resistant stock colony (showing recessive, monogenic resistance to Cry1Ac toxins) [58] were crossed to 25 females from the homozygous-susceptible ‘Geneva 88’ colony; 59 of the F1 male progeny from this cross were then mated to 100 females from the Geneva 88 strain. A total of 250 males and 250 females from this cross were then mated to produce the founder strain. These crosses provide an expected resistance allele frequency of 0.25 and an expected homozygous-resistant frequency of 0.0625. At all stages of founder strain production and maintenance, progeny were reared on wild-type broccoli plants in large numbers (>500 pupae collected per generation) to minimize the effects of inbreeding and genetic drift. However, *Bt* survival assay results (Fig. 2b) suggest that this frequency had increased to 0.36 (r = √0.1265) by the start of the experiment.

### Experimental broccoli cultivars

Two strains of broccoli (*Brassica oleracea* L.) plants were used: a wild-type cultivar (Packman) and a transgenic strain engineered to express high levels of the *Bt* toxin Cry1-Ac [41]. Together, this transgenic plant cultivar and the Cry1-Ac-resistant *P. xylostella* strain comprise a well-established model system used to study the dynamics of transgenic crops and their resistance management [58, 59]. Cry1-Ac toxin production was verified by screening the plants (4–5 weeks old) with susceptible Geneva 88 strain neonates. Leaf assays of transgenic plants also killed 100 % of OX4319L/Cry1-Ac hybrids, indicating high levels of *Bt* toxin expression.

### Restocking of plant material

All cages started with 20 broccoli plants of their respective cultivar. Plants were replaced after 4 weeks, or when defoliated due to larval feeding, by cutting them at their base and placing them on the replacement plants to allow larvae to transfer. If moth populations grew beyond the capacity of the maximum food supply (estimated by exceeding their cage’s plant material within one generation), the cage was terminated.

### Establishment of *P. xylostella* populations in cages

In all treatments, replicates were initiated by the release of *P. xylostella* adults from the founder strain into the cages. In treatments involving *Bt* plants, 200 randomly selected adults were released. In treatments involving non-*Bt* plants, seven males and females (total 14 adults) were released into the cages. Due to *Bt* selection, this gave approximately equal starting population densities in Generation 1 in all cages.

### Male-selecting transgenic insect treatments

OX4319L-homozygous males for release into the cages were produced by rearing egg collections from a stock colony in the absence of tetracycline in larval feed. Releases in this experiment were proportional: daily estimates of adult male recruitment for each cage were used to calculate a daily male release number for that cage, dependent on the release rate pre-determined for that treatment. A proportional release rate, rather than a constant number, was applied to reduce the likelihood of population extinction and thereby allow exploration of the effect of MS transgene releases on population dynamics and resistance allele frequency.

Release rates were selected in advance based on the outcomes of predictive deterministic models, investigating the effects of each experimental treatment on population size.

In Treatments 2 and 3 (low-OX4319L release treatments), the intended over-flooding ratio was 5:1 (transgenic: wild-type males). However, due to insect rearing limitations this was limited to 3:1 in Generation 1, and increased to 5:1 thereafter. These low release rates, in combination with *Bt* plants, were predicted to maintain a relatively constant population size, but insufficient to cause population suppression when used alone. Similarly, the over-flooding rates in cages with high-OX4319L releases, initially 20:1 and increased to 40:1 in Generation 2 as production capacity increased, were predicted to be sufficient for population suppression when used alone.

Where small populations are expanding in the absence of limiting biotic or abiotic factors, stochastic effects make it difficult to accurately predict the rate of population increase. In response to higher-than-predicted population growth and limited plants available, the cages with *Bt* broccoli plants and low-rate weekly OX4319L releases were reduced to two replicates in Generation 3 (terminated cage selected at random). In Generation 4, the number of plants in each treatment with *Bt* broccoli only (no OX4319L releases) cage was reduced to five (while maintaining per-plant population density levels) in response to limited plant availability. This was achieved by randomly harvesting 25 % of the leaves on each plant within the cage, removing all insect and plant material from the cage, and then restocking the cage with five new plants and returning harvested leaves. As generations were still discrete, this manipulation was timed during a period when the vast majority of insects in the population were present as larvae or pupae on plants, allowing accurate culling of the population. In Generation 3, requirements for OX4319L male moths exceeded production capacity, so the treatment with high-rate weekly OX4319L releases was reduced to one replicate only.

### Data collection

#### Population size estimation

The numbers of second to fourth instar larvae and pupae on each plant in each cage were counted once per week.

#### Bt resistance assays

With the exception of cages with high-rate OX4319L releases, *Bt* resistance data were collected from all cages in their final generation. As each cage reached maximum egg-laying potential (judged by female recruitment data in each population, collected from eclosion cages), eggs were collected from 8–10 leaves selected at random from each cage. These eggs were transferred to filter paper by paintbrush and resulting larvae were reared on chlortetracycline hydrochloride-augmented artificial diet (100 μg/mL). For each cage, two replicated *Bt* survival assays and one control assay were performed. For *Bt* assays, chlortetracycline hydrochloride-augmented diet was poured into 30 mL plastic pots and 500 μL of 10 ppm *Bt* (Dipel®, Valent BioSciences Corp., Libertyville, IL) – shown previously to discriminate between homozygous-resistant and other genotypes [46] – pipetted onto the dried surface. Non-*Bt* controls were prepared in the same way, with no added *Bt* solution. At third instar, larvae were transferred onto the air-dried diet surface. Exact numbers of larvae per replicate differed between cages according to availability of eggs. A minimum of 33 larvae per pot were used for the first *Bt* replicate; 11 per pot for the second *Bt* replicate; and all control replicates contained 20 larvae. Mortality was assessed 72 h later with surviving larvae taken to be homozygous for the resistance allele. For comparison, the founder strain was subjected to the same assay, in the generation prior to experimental initiation.

#### Bt resistance allele frequency estimation

*Bt* assay survival data from the founder strain, *Bt* broccoli alone, and low OX4319L + *Bt* broccoli treatments were used to calculate frequency of the *Bt* resistance allele, as conducted in previous studies utilizing this model system [46, 47, 58]. Under strong *Bt* selection (preliminary assays showed 100 % *Bt* susceptibility in homozygous susceptible and OX4319L/resistant heterozygotes) in the absence of resistance management efforts (as in the *Bt* broccoli-only treatment) it was expected that the *Bt* resistance allele would reach fixation from Generation 1 onwards (all individuals homozygous resistant). Deviation from 100 % *Bt* assay survival in this treatment thus represents mortality to a small proportion of homozygous-resistant individuals under these conditions. This reduction in survival was used to calibrate the results of the assay for other treatments prior to calculation of allele frequencies. Once calibrated, the square of the proportion surviving gave an estimate of the *Bt* allele frequency in the founder strain (under Hardy-Weinberg equilibrium). In the low OX4319L + *Bt* broccoli treatment, the calibrated survival rate represents the proportion of homozygous resistant individuals in the population, with the remainder of (non-surviving) individuals assumed to be the (heterozygous) offspring of resistant females and OX4319L males (as the only source of susceptibility alleles in these populations). The validity of this assumption was explored by comparing the percentage of non-surviving individuals with DsRed2 fluorescence proportions. If the mechanism of resistance management proposed in this experiment were functioning as predicted (released MS males providing susceptibility alleles through mating, maintaining *Bt* efficacy), we would expect to see high levels of agreement between population-level *Bt* susceptibility and fluorescence proportions in the low OX4319L + *Bt* broccoli treatment (where OX4319L males were hypothesized to be the sole providers of susceptibility alleles) but no such agreement in the low OX4319L only treatment populations, which received identical doses of MS transgenic males (and thus would be expected to show similar fluorescence proportions to the combined OX4319L + *Bt* treatment) but where *Bt* resistance alleles were not under selection. The *Bt* resistance allele frequency in the low OX4319L + *Bt* broccoli treatment was calculated as [Χ + 0.5(1–Χ)], where Χ is the calibrated survival rate.

#### Fluorescence proportion assays

The population-level fluorescence proportion (proportion of the population carrying the transgene) was estimated for all cages into which OX4319L males were released, in the final generations they were conducted. The exception to this was the high OX4319L release treatment where this assay was conducted in the penultimate (third) generation due to the small population size of this treatment cage in Generation 4. Collection of eggs followed procedures described for *Bt* resistance assays with larvae being reared to pupation on chlortetracycline hydrochloride-augmented artificial diet (100 μg/mL) in 500 mL Styrofoam pots. At pupation, individuals were screened for presence/absence of the DsRed2 fluorescent protein marker. Each cage acted as a replicate with a minimum of 75 larvae per assay pot.

#### Male recruitment rate estimation

For those cages requiring OX4319L male introductions, daily male recruitment rate was assessed by the removal of four randomly selected plants/cage/week. These were placed into smaller cages in an adjacent glasshouse maintained in the same environmental conditions. The number and sex ratio of the adults eclosing from these plants were recorded daily, and eclosed adults were then returned to their respective experimental cages. From this data an estimate of the eclosion rates in the main cage was made, from which the number of OX4319L males required to achieve the respective over-flooding ratio for each cage was calculated.

### Statistical analyses

For comparisons between treatment peak population densities in Generation 3, ANOVA was used, followed by Tukey’s HSD for pair-wise comparisons. For Generation 4, a *t*-test was used as only two treatments were compared. For *Bt* assay results, results from the two *Bt* assays within each cage were summed to avoid pseudo-replication and then corrected for control mortality using a Henderson-Tilton correction. For comparisons between treatments, a Logit model for Categorical Data Analysis was used followed by pair-wise comparisons using Generalized Linear Hypothesis Testing with each cage acting as a replicate. Error estimates for mean proportions were calculated using Pearson’s exact confidence intervals. Data were analyzed using R (Version 2.15.0).

## Abbreviations

*Bt*: *Bacillus thuringiensis*
IPM: Integrated pest management
IRM: Insecticide resistance management
MS: Male-selecting
SIT: Sterile insect technique

## References

[1] Lounibos LP (2002). Invasions by insect vectors of human disease. Annu Rev Entomol..

[2] Kenis M, Auger-Rozenberg M-A, Roques A, Timms L, Péré C, Cock M (2009). Ecological effects of invasive alien insects. J Biol Inv..

[3] Oerke E-C (2006). Crop losses to pests. J Agric Sci..

[4] Knipling E (1955). Possibilities of insect control or eradication through the use of sexually sterile males. J Econ Entomol..

[5] Dyck VA, Hendrichs J, Robinson AS (2005). Sterile insect technique: principles and practice in area-wide integrated pest management.

[6] Bakri A, Mehta K, Lance DR, Dyck VA, Hendrichs J, Robinson AS (2005). Sterilizing insects with ionizing radiation. Sterile insect technique principles and practice in area-wide integrated pest management.

[7] Rull J, Brunel O, Mendez M (2005). Mass rearing history negatively affects mating success of male *Anastrepha ludens* (Diptera: Tephritidae) reared for sterile insect technique programs. J Econ Entomol..

[8] Davich T, Lindquist D (1962). Exploratory studies on gamma radiation for the sterilization of the boll weevil. J Econ Entomol..

[9] Sharma V, Razdan R, Ansari M (1978). *Anopheles stephensi*: effect of gamma-radiation and chemosterilants on the fertility and fitness of males for sterile male releases. J Econ Entomol..

[10] Helinski M, Knols B (2008). Mating competitiveness of male *Anopheles arabiensis* mosquitoes irradiated with a semi- or fully-sterilizing dose in small and large laboratory cages. J Med Entomol..

[11] Hagler J, Jackson C (2001). Methods for marking insects: current techniques and future prospects. Annu Rev Entomol..

[12] Hagler JR, Miller E (2002). An alternative to conventional insect marking techniques: detection of a protein mark of pink bollworm by ELISA. Entomol Exp et Appl..

[13] Robinson AS, Hendrichs J, Dyck VA, Hendrichs J, Robinson AS (2005). Prospects for the future development and application of the sterile insect technique. Sterile insect technique principles and practice in area-wide integrated pest management.

[14] Thomas DD, Donnelly CA, Wood RJ, Alphey LS (2000). Insect population control using a dominant, repressible, lethal genetic system. Science..

[15] Jin L, Walker AS, Fu G, Harvey-Samuel T, Dafa'alla T, Miles A (2013). Engineered female-specific lethality for control of pest Lepidoptera. ACS Syn Biol..

[16] Fu G, Condon KC, Epton MJ, Gong P, Jin L, Condon GC (2007). Female-specific insect lethality engineered using alternative splicing. Nat Biotechnol..

[17] Fu G, Lees RS, Nimmo D, Aw D, Jin L, Gray P (2010). Female-specific flightless phenotype for mosquito control. Proc Natl Acad Sci U S A..

[18] Ant T, Koukidou M, Rempoulakis P, Gong HF, Economopoulos A, Vontas J (2012). Control of the olive fruit fly using genetics-enhanced sterile insect technique. BMC Biol..

[19] Schliekelman P, Gould F (2000). Pest control by the release of insects carrying a female-killing allele on multiple loci. J Econ Entomol..

[20] Leftwich PT, Koukidou M, Rempoulakis P, Gong HF, Zacharopoulou A, Fu G, et al. Genetic elimination of field-cage populations of Mediterranean fruit flies. Proc Biol Sci. 2014;281(1792). 10.1098/rspb.2014.1372 PMC4150327 25122230

[21] Walters M, Morrison NI, Claus J, Tang G, Phillips CE, Young R (2012). Field longevity of a fluorescent protein marker in an engineered strain of the pink bollworm, *Pectinophora gossypiella* (Saunders). PLoS One..

[22] Rendón P, McInnis D, Lance D, Stewart J, Tan K (2000). Comparison of medfly male-only and bisexual releases in large scale field trials. Area-wide control of fruit flies and other insect pests.

[23] Rendón P, McInnis D, Lance D, Stewart J (2004). Medfly (Diptera:Tephritidae) genetic sexing: large-scale field comparison of males-only and bisexual sterile fly releases in Guatemala. J Econ Entomol..

[24] Franz G, Dyck VA, Hendrichs J, Robinson AS (2005). Genetic sexing strains in Mediterranean fruit fly, an example for other species amenable to large-scale rearing for the sterile insect technique. Sterile insect technique principles and practice in area-wide integrated pest management.

[25] Cáceres C (2002). Mass rearing of temperature sensitive genetic sexing strains in the Mediterranean fruit fly (*Ceratitis capitata*). Genetica..

[26] Robinson A, Franz G, Fisher K (1999). Genetic sexing strains in the medfly, *Ceratitis capitata*: development, mass rearing and field application. Trends Entomol..

[27] de Valdez MRW, Nimmo D, Betz J, Gong HF, James AA, Alphey L (2011). Genetic elimination of dengue vector mosquitoes. Proc Natl Acad Sci U S A..

[28] Bloem KA, Bloem S, Carpenter JE, Dyck VA, Hendrichs J, Robinson AS (2005). Impact of moth suppression/eradication programmes using the sterile insect technique or inherited sterility. Sterile insect technique principles and practice in area-wide integrated pest management.

[29] Alphey N, Bonsall M, Alphey L (2009). Combining pest control and resistance management: synergy of engineered insects with *Bt* crops. J Econ Entomol..

[30] Alphey N, Coleman PG, Donnelly CA, Alphey L (2007). Managing insecticide resistance by mass release of engineered insects. J Econ Entomol..

[31] James C (2013). Global status of commercialized biotech/GM crops. ISAAA Briefs.

[32] Tabashnik BE, Brevault T, Carriere Y (2013). Insect resistance to Bt crops: lessons from the first billion acres. Nat Biotechnol..

[33] James C (2014). Global status of commercialized transgenic crops. ISAAA Briefs.

[34] Bates SL, Zhao JZ, Roush RT, Shelton AM (2005). Insect resistance management in GM crops: past, present and future. Nat Biotechnol..

[35] Tabashnik BE, Gould F (2012). Delaying corn rootworm resistance to *Bt* corn. J Econ Entomol..

[36] Zalucki MP, Shabbir A, Silva R, Adamson D, Shu-Sheng L, Furlong MJ (2012). Estimating the economic cost of one of the world's major insect pests, *Plutella xylostella* (Lepidoptera: Plutellidae): just how long is a piece of string?. J Econ Entomol..

[37] Tabashnik B, Cushing N, Finson N, Johnson M (1990). Field development of resistance to *Bacillus thuringiensis* in diamondback moth (Lepidoptera: Plutellidae). J Econ Entomol..

[38] Shelton A, Robertson J, Tang J, Perez C, Eigenbrode S, Preisler H (1993). Resistance of diamondback moth (Lepidoptera: Plutellidae) to *Bacillus thuringiensis* subspecies in the field. J Econ Entomol..

[39] Whalon M, Mota-Sanchez D, Hollingworth R (2008). Global pesticide resistance in arthropods.

[40] Furlong M, Wright D, Dosdall L (2013). Diamondback moth ecology and management: problems, progress, and prospects. Annu Rev Entomol..

[41] Metz TD, Roush RT, Tang JD, Shelton AM, Earle ED (1995). Transgenic broccoli expressing a *Bacillus thuringiensis* insecticidal crystal protein: Implications for pest resistance management strategies. Mol Breed..

[42] Cao J, Shelton AM, Earle ED (2008). Sequential transformation to pyramid two Bt genes in vegetable Indian mustard (*Brassica juncea* L.) and its potential for control of diamondback moth larvae. Plant Cell Report.

[43] Cao J, Shelton A, Earle ED (2005). Development of transgenic collards expressing a cry1Ac or cry1C Bt gene for control of the diamondback moth. Crop Protect.

[44] Cao J, Zhao J, Tang JD, Shelton A, Earle ED (2002). Broccoli plants with pyramided *cry1Ac* and *cry1C* Bt genes control diamondback moths resistant to Cry1A and Cry1C proteins. Theor Appl Genet..

[45] Cao J, Tang JD, Strizhov N, Shelton A, Earle ED (1999). Transgenic broccoli with high levels of *Bacillus thuringiensis* Cry1C protein control diamondback moth larvae resistant to Cry1A or Cry1C. Mol Breed..

[46] Shelton AM, Tang JD, Roush RT, Metz TD, Earle ED (2000). Field tests on managing resistance to Bt-engineered plants. Nat Biotechnol..

[47] Tang JD, Collins HL, Metz TD, Earle ED, Zhao J, Roush RT (2001). Greenhouse tests on resistance management of Bt transgenic plants using refuge strategies. J Econ Entomol..

[48] Harvey-Samuel T, Ant T, Gong HF, Morrison NI, Alphey L (2014). Population-level effects of fitness costs associated with repressible female-lethal transgene insertions in two pest insects. Evol Appl..

[49] Harcourt D, Talekar N (1986). Population dynamics of the diamondback moth in southern Ontario. Diamondback moth and other crucifer pests, First International Workshop: 11–15 March 1985.

[50] Smith D, Sears M (1982). Evidence for dispersal of diamondback moth, *Plutella xylostella* (Lepidoptera: Plutellidae), into southern Ontario. Proc Entomol Soc Ont..

[51] Honda K, Talekar NS (1992). Hibernation and migration of the diamondback moth in north Japan. Management of Diamondback Moth and Other Crucifer Pests: Proceedings of the Second International Workshop.

[52] Chapman J, Reynolds DS, Riley J, Pedgley D, Woiwod I (2002). High-altitude migration of the diamondback moth, *Plutella xylostella*, to the UK: a study using radar, aerial netting and ground trapping. Ecol Entomol..

[53] Mo J, Baker G, Keller M, Roush R (2003). Local dispersal of the diamondback moth (*Plutella xylostella* (L.)) (Lepidoptera: Plutellidae). Environ Entomol.

[54] Cameron P, Walker G, Penny G, Wigley P (2002). Movement of potato tuberworm (Lepidoptera: Gelechiidae) within and between crops, and some comparisons with diamondback moth (Lepidoptera: Plutellidae). Popul Ecol..

[55] Bariola L, Keller J, Turley D, Farris J (1973). Migration and population studies of the pink bollworm in the arid West. Environ Entomol..

[56] Mani E, Wildbolz T (1977). The dispersal of male codling moths (*Laspeyresia pomonella* L.) in the Upper Rhine Valley. Zeitschrift für Angewandte Entomologie.

[57] Knipling E (1979). Agriculture Handbook no. 512: The basic principles of insect population suppression and management.

[58] Zhao JZ, Cao J, Li Y, Collins HL, Roush RT, Earle ED (2003). Transgenic plants expressing two *Bacillus thuringiensis* toxins delay insect resistance evolution. Nat Biotechnol..

[59] Zhao JZ, Cao J, Collins HL, Bates SL, Roush RT, Earle ED (2005). Concurrent use of transgenic plants expressing a single and two *Bacillus thuringiensis* genes speeds insect adaptation to pyramided plants. Proc Natl Acad Sci U S A..

[60] Liu X, Chen M, Collins HL, Onstad DW, Roush RT, Zhang Q (2014). Natural enemies delay insect resistance to *Bt* crops. PLoS One..

[61] Tabashnik BE, Sisterson MS, Ellsworth PC, Dennehy TJ, Antilla L, Liesner L (2010). Suppressing resistance to *Bt* cotton with sterile insect releases. Nat Biotechnol..

[62] Johnson M, Gould F, Kennedy G (1997). Effect of an entomopathogen on adaptation of *Heliothis virescens* populations to transgenic host plants. Entomol Exp Appl..

[63] Onstad DW, Liu X, Chen M, Roush R, Shelton A (2013). Modeling the integration of parasitoid, insecticide and transgenic insecticidal crops for the long-term control of an insect pest. J Econ Entomol..

[64] Bommarco R, Miranda F, Bylund H, Björkman C (2011). Insecticides suppress natural enemies and increase pest damage in cabbage. J Econ Entomol..

[65] Hallmann CA, Foppen RP, van Turnhout CA, de Kroon H, Jongejans E (2014). Declines in insectivorous birds are associated with high neonicotinoid concentrations. Nature..

[66] Tan A, Fu G, Jin L, Guo Q, Li Z, Niu B (2013). Transgene-based, female-specific lethality system for genetic sexing of the silkworm, *Bombyx mori*. Proc Natl Acad Sci U S A..

[67] Martins S, Naish N, Walker AS, Morrison NI, Scaife S, Fu G (2012). Germline transformation of the diamondback moth, *Plutella xylostella* L., using the *piggyBac* transposable element. Insect Mol Biol.

[68] Walters M, Staten R, Roberson R, Tan K (2000). Pink bollworm integrated management using sterile insects under field trial conditions, Imperial Valley, California. International Conference on Area-Wide Control of Insect Pests, and the 5th International Symposium on Fruit Flies of Economic Importance: 28 May - 5 June 1998.

[69] Proverbs M, Newton J, Campbell C (1982). Codling moth: a pilot program of control by sterile insect release in British Colombia. Can Entomol..

[70] Suckling D, Hackett J, Barrington A, Daly J (2002). Sterilisation of painted apple moth *Teia anartoides* (Lepidoptera: Lymantriidae) by irradiation. N Z Plant Protect..

[71] Wee S, Suckling D, Burnip G, Hackett J, Barrington A, Pedley R (2005). Effects of substerilizing doses of gamma radiation on adult longevity and level of inherited sterility in *Teia anartoides* (Lepidoptera: Lymantriidae). J Econ Entomol..

